# Patterns of Use of an Automated Interactive Personalized Coaching Program for Smoking Cessation

**DOI:** 10.2196/jmir.1016

**Published:** 2008-12-17

**Authors:** James Balmford, Ron Borland, Peter Benda

**Affiliations:** ^2^Department of Information SystemsUniversity of MelbourneVictoriaAustralia; ^1^VicHealth Centre for Tobacco ControlThe Cancer Council VictoriaCarltonVictoriaAustralia

**Keywords:** Smoking cessation, Internet, behavioral medicine

## Abstract

**Background:**

The QuitCoach, an “expert system” program of tailored advice for smoking cessation developed in Australia, has been publicly available since July 2003, albeit with limited promotion. The program is designed to be used on multiple occasions, guiding the user through the process of smoking cessation in the manner of a “life coach”. Email reminders are sent at scheduled intervals to prompt optimal and repeated use.

**Objectives:**

The aims of this study were to characterize QuitCoach users and to determine what characteristics of smokers affect their participation over time. Of particular interest was whether users tend to return following a relapse and, thus, use the program as a tool for relapse prevention or recovery. We also explored patterns of change associated with returns to the site, whether prompted by reminder emails or not prompted at all.

**Methods:**

Between July 2003 and June 2007, 28,247 individuals completed an initial assessment on the QuitCoach, of whom 83.7% (n = 23,656) registered. Data were collected during a 10-minute online questionnaire that all users completed in order to obtain tailored cessation advice. This included questions concerning basic demographic information, quitting history, current smoking status and cigarette consumption, stage of change, and use of pharmacotherapy.

**Results:**

The median age of users was 34 years, and 62% were female. Most (96%) were current smokers. Overall, 91% were planning to quit in the next 30 days, and half (49.9%) had set a quit date. Those who had recently relapsed to smoking following a quit attempt made up 37%. Among registered users, 27% returned for a second visit, a median 9 days after their first. Overall, a third visit was completed by 11% and 2% returned within 2 days. Women, older smokers, those who had recently quit, and those using pharmacotherapy were more likely to return. From the second visit on, most people who completed an assessment had quit. Likelihood of responding to a prompt to return was largely unrelated to user characteristics or cessation outcome.

**Conclusions:**

Internet-based programs have considerable potential to reach large numbers of smokers at low cost. The QuitCoach is attracting considerable use, with most using it to make a quit attempt and, for those who continue to use the QuitCoach, to help them stay quit. Nonetheless, most users only visited the site once, suggesting improved strategies are needed for encouraging repeated use.

## Introduction

Cigarette smoking is a major public health problem. Worldwide, tobacco use claims an estimated 4.9 million lives annually [1], with this figure expected to increase to 10 million per year by 2030 [2]. Peto et al [2] have noted that helping current smokers to quit is the single most important step to reduce mortality and morbidity associated with cigarette smoking in the short term.

Many smokers find it difficult to quit. While most smokers have tried, only 3 -5% are able to achieve prolonged abstinence for 6 - 12 months after a given unassisted quit attempt [3]. It is clear, however, that good quality structured support and advice increases smoking cessation rates over self-managed attempts, with long-term success rates reaching approximately 15 - 20% independent of any effect attributable to use of pharmacotherapy [4].

Computer-generated advice programs, which can provide personalized smoking cessation advice tailored to the characteristics of individual smokers, are a promising medium for delivering effective smoking cessation assistance to large numbers of smokers [5]. A recent meta-analysis found that tailored advice is more effective than generic (non-tailored) advice, despite considerable variation in the quality of tailored programs that have been tested in trials [6]. Tailored advice programs that have the capacity to monitor progress over time in a timely fashion and provide feedback in response to changes (ie, build an ongoing relationship with the smoker through their quit attempt) are likely to be most effective [7].

In this paper, we explore how QuitCoach [8], a tailored, automated advice program program, is used, and which characteristics of smokers affect usage patterns.

## Methods

### The QuitCoach

The QuitCoach [8] is the tailored, automated advice program which is the focus of this paper. It was developed at The Cancer Council Victoria, Australia, and is provided by Quit Victoria. The site is currently promoted quite widely on Australian quit smoking websites, including QuitNow [9], the website on all Australian cigarette packs since 2006, and Quit [10], Quit Victoria’s smoking cessation site.

The QuitCoach is designed to be used on multiple occasions, guiding the user through the process of smoking cessation in the manner of a “life coach”. It is designed to provide tailored advice replicating many of the core features of in-person, multi-session cognitive-behavioral counseling. Following an online assessment (typically 10 - 15 minutes in duration), users receive advice tailored to the answers they provided. The advice is based on an integrated mix of empirically grounded modifications to stage-based and other cognitive-behavioral theories of behavior change [11], focusing on behavioral strategies, cognitive restructuring, and motivation. Particular attention has been paid to relapse prevention, using a model that explicitly takes into account discontinuity in the proximal task associated with quitting over time [12].

The advice is organized around what we call perspectives on change, which are revisions of the stages of change of Prochaska and associates [13]. We call them perspectives because they represent different points of view from which the quitting process is experienced by the people taking themselves through the process [14]. The perspectives and the labels we give them here are specified in Table 1. The critical transitions defining perspective boundaries pre-quitting are: deciding to think actively about quitting or planning to quit; setting an implementation plan (eg, a quit date); and actually quitting. Post-quitting, there are less clear transitions between the first few days, when withdrawal symptoms are likely to be highest, and the subsequent period, separated by the time when the frequency of strong urges to smoke drops below daily without pharmacological assistance. The perspectives were derived both from conceptual analysis of the process of smoking cessation and from empirical evidence of discontinuity in predictive capacity across the boundaries [12]. This distinguishes the perspectives on change from the arbitrarily defined boundaries postulated by Prochaska [15].

**Table 1:** Perspectives on change and their definition

Perspective	Definition
Not planning	Not planning to quit in the next 30 days.
Planning	Planning to quit in the next 30 days, but not meeting criteria for committed perspective.
Set date	Setting a quit date in the next 2 weeks or cutting down to quit and expects to cut down to zero within 2 weeks.
Just quit	First week of quit attempt, or up to 2 weeks if cravings are described as continual (hourly or more often).
Consolidation	From end of “just quit” till strong urges to smoke occur less than daily, or concurrent use of quit smoking medication.
Established	Less than daily strong urges to smoke and no use of quit smoking medication.

The advice provided is individually tailored in response to answers to questions asked in a set of 5 modules. To receive tailored advice, a user needed only to complete the first “core” module. Following this, users had the option of viewing their tailored advice or continuing on to complete other modules. Following the completion of each module, more detailed advice was appended to the advice document. The core module covered demographics and smoking history (both largely only in the initial assessment), and at each assessment the following were covered: current smoking patterns, recent quitting activity, use of or plans to use cessation help, assessment of perspective on change, and affect. The second module, also asked only in the initial assessment, included questions on the user’s social context: household composition, presence of smoking bans at home and at work, proportion of friends who smoke, social support to quit, and medical conditions relevant to quitting. The three additional modules assess perceived values of smoking, reasons for quitting and perceived barriers, self-efficacy, and processes of change for quitting. Tailored advice is provided on all these topics, identifying strengths and areas where extra work is likely needed to progress.

If users have registered on the site, they can use it to update assessments and, thus, the advice provided to them. In subsequent visits, the questions asked are only those relevant to the person’s new situation, and the advice provided takes into account changes the user has made since the previous visit, as well as their situation at the time. The focus of change is generally restricted to the implications of change between perspectives, when such shifts occur. When the person remains within a pre-quit perspective, changes in other variables are used to help diagnose problems that are preventing them from moving forward and to offer possible solutions. Post-quitting, progress in reduction of urges to smoke and beliefs about the need to smoke in various contexts is tracked and, thus, progress to becoming a stable non-smoker. Progress is congratulated and areas of inadequate progress or regress analyzed, and recommendations are provided for increased focus on strategies for overcoming the identified problems.

Registered users are encouraged to return when their situation changes. In addition, they are sent a reminder email to log-in on a follow-up date scheduled by the program, based on an algorithm designed around their perspective on change, with returns encouraged more rapidly for those closest to their quit date. Therefore, there can be considerable variation in the time from an assessment to when an email is sent to encourage a new one. A second, and final, reminder is sent several days after the first, if the user has not logged on in the meantime.

A prototype version of the QuitCoach, in which users were telephoned for each assessment and mailed the tailored advice (fundamentally identical to the website) was demonstrated in a randomized trial to be effective in a sample of smokers seeking help [14]. Using a conservative analysis (in which missing cases at follow-up were treated as smokers), 20% of those who received the program achieved 6-month sustained abstinence at 12-month follow-up, compared with 12% in a control group receiving one-off standard printed self-help materials (OR 1.82, 95% CI 1.31 - 2.55). The effect size was comparable with typical effects of face-to-face cessation counseling [16].

The effectiveness of the program in the RCT is likely to have been enhanced by high levels of optimal participation. With repeat visits prompted by a telephone call, over half of trial participants accepted five or more visits. Ongoing use of the program was related to greater likelihood of success [14]. However, as an Internet resource in which users are prompted to return by email rather than a telephone call, most users only use it once, and only around 10% use it more than twice [17].

Nonetheless, email reminders consisting of a simple message to return to the site for updated advice appear to stimulate return [17]. Returns to the site were generally clustered around the times the emails were sent out. However, as most users failed to return at all, their effectiveness is clearly limited. Graham et al [17] also found that a significant minority of users returned on the same day, or the day after, their first visit.

As compared with smokers in general, QuitCoach users are more likely to be female, aged 25 - 44, and more nicotine dependent. Compared with users of a Quitline, they are also more likely to be female, aged 20 - 49, somewhat less dependent, and less likely to have already started their quit attempt [18].

### Participants

The study covers the period from January 2003, when the site first became available to the public, to June 2007, when the site was replaced by a new version. There were 29,524 separate records in the QuitCoach user database (excluding 285 test cases). Over the last 5 months of use (January - May 2007), new users were completing assessments at an average rate of almost 900 per month. 

At the beginning of the core assessment, participants were asked to complete the following statement: “I am using the QuitCoach because...”. Of the respondents, 83.8% (n = 24,740) indicated they were using the QuitCoach because they wanted advice to help them quit smoking or stay quit; 12.2% (n = 3594) were “just having a look”; and 3.5% (n = 1048) indicated they were a health professional or researcher interested in the way the program worked. A few (n = 142) respondents who gave other reasons for using the website, for example health professionals and a subset of ex-smokers (more than 6 months quit who were “just having a look”), were excluded from further analysis. The final eligible sample was 28,247 users.

### Measures

All data were collected during the standard QuitCoach smoking assessment [14].

The information used in this paper comes primarily from the core module at the initial assessment (visit 1) which was completed by all users whose data was retained. The data used included a person’s gender and age, perspective on change (Table 1), recent quitting history, reported use of pharmacotherapy (eg, nicotine replacement therapy, bupropion), use of other professional cessation assistance, and dependence as measured using the heaviness of smoking index—alternate version (HSI-AV) [19], calculated as the square root of daily cigarette consumption minus the natural logarithm of time to first cigarette of the day. At each subsequent visit, we used data on current perspective on change and recent quitting history. In addition, data was collected on registration status, number of modules completed at each visit, time between visits, and number of visits.

Three kinds of basic usage were defined: to make a quit attempt (all smokers using who had not relapsed in the last week); to recover from relapse (smokers relapsing in the last week); and to sustain a quit attempt (all using when quit). Returns to the site were coded as follows: before a scheduled prompt to return was sent; from the initial prompt to just before the second prompt; from the second prompt for 1 week; and any later return. The actual duration of these categories varied by perspective on change, with those closest to the point of quitting receiving their first scheduled reminder email earlier than those further from quitting. For those in the “Not planning” and “Established” perspectives, the first reminder was sent 30 days after the initial visit; for those in “Planning” and “Consolidation”, the interval was 2 weeks; and for those “Just quit”, it was 1 week. For those who had set a quit date, the email was sent 4 days after their quit date.

### Statistical Analysis

Descriptive statistics were used to characterize the sample. Differences between groups were determined using the Pearson’s chi-square test for categorical variables and the independent sample *t* test for continuous variables. For non-normally distributed variables we report the inter-quartile range (IQR). An alpha level of *P* < .01 was used for all statistical tests, given the large sample size.

## Results

### User Profile

Users were predominantly female (62%). The median age was 34 years, with 16.6% ≤ 25 years, 37% aged 26 - 35, 33.5% aged 36 - 49, 11.4% aged 50 - 64, and 1.6% aged over 65 years. Users were a median 17 (IQR = 15 - 18) years old when they first started to smoke daily and had been smoking cigarettes for a median 17 (IQR = 9 - 25) years. Current smokers smoked an average of 19 (SD = 9.7) cigarettes per day, with their first cigarette of the day a median 20 (IQR = 10 - 60) minutes after waking, and nearly all (98.5%) smoked daily.

Most users (95.8%) were current smokers at their first visit. Of these, 4.5% were not planning a quit attempt, 41.4% were planning without a set date, and 49.9% had set a quit date or a cut down schedule. The 4.2% of quitters consisted of 1.6% recent quitters (within the last week), 2.0% consolidating (quit more than a week ago but with daily urges to smoke and/or current use of quit smoking medication), and 0.7% established (reporting less than daily urges and no use of medication). Men who visited were slightly more likely than women to visit as smokers before setting a quit date. A similar pattern was seen for the youngest (< 25) and older (> 50) age groups.

Among those smoking at baseline, 12.2% had not previously tried to quit, 11.9% had not quit (for more than 24 hours) in the last 5 years, 26.6% had last tried 1 - 5 years ago, 30.1% 1 month to 1 year ago, 7.5% 1 week to 1 month ago, and 11.7% had tried in the last week (including 2.7% who had relapsed earlier on the day of the initial QuitCoach visit). Of those who had tried to quit in the last 5 years, 16.6% had a longest attempt of less than a week, 20.1% from 1 week to 1 month, 30.5% from 1 month to a year, and 8.6% for a year or more.

Among those quit at baseline, 36.9% had been quit for less than a week, including 7.9% who had quit “today”, and 11.6% who had been quit for only 1 or 2 days. A further 36.2% had been quit from 1 week to 1 month.

Overall, 22.6% (n = 6371) of the sample reported currently using pharmacological support, with 83.3% using some form of nicotine replacement (NRT, patch, gum, lozenge, or inhaler) and 15.1% using bupropion. Other professional help was being sought by 3.4% (n = 916) of users. Of these, quit counseling (n = 418) and advice from a doctor/psychiatrist (n = 331) were the most frequently accessed forms of help. A further 4.5% of users (n = 1208) reported that they were getting help from friends, family, or a self-help manual.

Most users (60.9%) completed all five of the question modules on their first visit. Those wanting advice to quit smoking (63.8%) were more likely to complete a full assessment than those “just having a look” (40.9%). Those planning to quit (61.9% of those just planning and 61.5% with a set quit date) were more likely to complete the full assessment than others. For example, only 53.3% of those already quit at baseline did so.

### Registration

Overall, 83.8% (n = 23,657) of users registered with the site. Participants who were “just having a look” were less likely to register (59.9%) than those who wanted advice to quit smoking (87.1%). Women were more likely to register (*P* < .001, see Table 2). Registration decreased linearly by age category (*P* < .001). Users in the set date and just quit perspectives (83.1%) were more likely to register than those in other perspectives (*P* < .001). 

Of those who completed the entire assessment at baseline, 91.8% registered, compared with 71.2% of those who only partially completed the assessment.

**Table 2:** Registration and return use of the site

Baseline Characteristics		% Registering	n	Returned once (n = 3747)	Returned more than once (n = 2714)	Total multiple use (n = 6460)
**Sex**						
	Male	81.6	8805	14.3	9.6	23.9
	Female	85.1	14837	16.8	12.5	29.3
**Age**						
	25 or under	88.9	4174	14.2	7.5	21.7
	26-35	87.5	9142	15.4	10.7	26.1
	36-49	80.8	7631	16.3	13.8	30.1
	50-64	74.7	2396	18.7	14.0	32.7
	65+	67.8	299	19.7	13.4	33.1
**Perspective on change**						
	Not planning	70.3	884	12.0	6.0	18.0
	Planning	81.0	9482	14.1	6.9	21.0
	Set date	87.5	12329	17.2	14.6	31.8
	Just quit	83.1	373	17.4	25.2	42.6
	Consolidation	80.3	449	22.9	20.3	43.2
	Established	73.3	140	15.0	17.1	32.1
**Previous attempt**						
	None	81.6	5141	13.7	9.5	23.2
	Short (< 1 wk)	84.0	3780	14.7	9.0	23.7
	Long (≧ 1 wk)	84.8	13769	16.7	12.2	28.1
**Recency of last quit**						
	< 1 week	84.5	2710	14.0	8.9	22.9
**Nicotine dependence (HSI-AV score)**						
	Less than 0	87.7	3329	15.1	9.2	24.3
	0 - < 2	85.2	6785	16.2	11.6	27.8
	2 - < 4	83.0	8310	15.5	11.4	26.9
	More than 4	80.9	4271	15.5	10.9	26.4
**Current use of pharmacotherapy**						
	Yes	86.0	5481	18.8	17.2	36.0
	No	83.1	18176	15.0	9.7	24.7
**Use of other professional help**						
	Yes	87.2	799	19.5	19.8	39.3
	No	84.3	21878	15.8	11.4	27.2
Total		83.8	23657	15.8	11.5	27.3
Note: All group comparisons were significant at the *P* < .001 level.						

### Repeated Use of the QuitCoach

Among registered users, 27.3% (n = 6461) returned for a second visit a median 9 (IQR = 6 - 15) days after their first visit. The time interval to first return varied from 0 (same day) to 365 days (NB cases were archived after one year of inactivity). Most of those who returned (73.0%) did so within 2 weeks, and 92.8% did so within a month. Notably, 20.3% of returns occurred before any prompt to return was sent (including 11.6% within 2 days). Most returns (56.9%) were in response to the initial prompt, and 14.2% returned up to one week after the second prompt, leaving 8.6% who returned of their own accord at some later time. The effect of prompting was unrelated to sex or age. Smokers not planning to quit at the initial assessment were unlikely to respond to either prompt (16.3% of those who returned cf 71.1% overall), being more likely to return for a second visit either before their initial prompt or after prompting had ended. Those with a set date were least likely to return early, but note they had less time to do so. 

The rate of return increased over time, with 31.1% of users post-June 2006 returning, compared with the 25.7% for pre-June 2006 users (*P* < .001). Three-quarters (74.1%) of those who returned for a second visit completed the full assessment at baseline, compared with only 64% of those who did not return.

Characteristics associated with repeat use are provided in Table 2. Women were more likely to return than men (*P* < .001), and returns increased linearly by age category (*P* < .001). In addition, those using pharmacotherapy (36.0%) were more likely to return, as were those seeking other professional help (39.3%). Return varied by baseline perspective (*P* < .001). It was highest among those who had just quit and in the consolidation perspective on change (42.6% and 43.2%, respectively) and lowest in those not planning an attempt. Among those with a quit date, likelihood of return was related to a recent relapse. Those who had recently relapsed to smoking (in the last week) were less likely to return (24.9%) than those without a recent failed attempt (*P* < .001).

### Progress While Using

Of the 6461 cases that returned for a second visit, 58.9% (n = 3808) were quit at their second visit, including 56.9% of the 6063 users who were smoking at the initial visit. A further 19.3% had made a quit attempt and subsequently relapsed, meaning that over three-quarters (76.2%) of smokers who returned for a second visit had made a quit attempt. Among those originally in the set date perspective (n = 3915), 90.9% at least made a quit attempt (72.9% quit, 18.0% relapsed). Those in the set date perspective who had recently relapsed (in the last week) at the initial visit were less likely to be quit at the second visit than those who had not (64.9% vs 73.8%, χ^2^_1_= 14.49, *P* < .001).

Those who were smoking at baseline and quit at second visit were quit a median 7 days (IQR = 6 - 14). Only 5.9% reported returning on the same day or the day after beginning their quit attempt. 

Most participants who were quit at baseline were still quit at their second visit (89.4%, n = 356), including 5% who had relapsed and then started a new quit attempt before returning.

Over a quarter (29.9%) of users who returned were using pharmacotherapy at their first visit, and of these 91.7% made a quit attempt by visit 2 compared with 70.1% of those not using medication. Of those who made a quit attempt, 81.4% of those using medication were quit at visit 2, compared with 70.5% of those not using medication. Uptake of medication from visit 1 to visit 2, which occurred for 20.9% of those not using, was associated with a quit attempt; 90.3% made a quit attempt compared with 64.7% who did not use medication at all. Of those making an attempt, 76.9% of those who took up medication were still quit at visit 2, compared to 68.2% of those not using medication.

Early returners (prior to the first email reminder) were less likely to have progressed in their quit attempts (43.4% had not made an attempt) compared with those responding to the prompts (17.3% for the initial prompt and 14.6% for the second one). However, those returning after prompting ended were also less likely to have made an attempt (32.4%). This latter group would appear to be users who had given up on their initial attempt and who had returned subsequently, presumably when they were more ready to try again.

We also explored whether those who returned very rapidly (within 2 days of the initial assessment) differed from the less rapid early returners. There were few notable differences, apart from the expected lower level of progression among the very early returns. A greater proportion of the early returns for those with a quit date were rapid, with those furthest from quitting least likely to return within 2 days (*P* < .001). Rapid returns also increased with increasing levels of dependence.

It is notable that 47.9% of those returning after prompting ended (4.1% of all second visits) are considering a new quit attempt. Only 15.4% were attempting to recover from a relapse. The remaining 36.7% were quit and were presumably using the program to overcome unexpected problems in maintaining their attempts. Among those returning either before or with prompting, only 3.7% (3.2% overall) could be considered to be initiating a new attempt, having relapsed back to smoking more than a week ago. Overall, 7.3% of returns were pursuing a subsequent quit attempt, 19.4% were continuing to pursue their initial attempt, 14.2% were recovering from a recent relapse, and 58.9% were using to stay quit (53.4% just quit and 5.5% quit before visit 1).

### Visit-by-Visit Progression

Patterns of outcomes for those returning to the site over the first 5 waves of data are summarized in Table 3. For each wave, among those who returned, we report status at their previous visit as well as at that visit to indicate what happened between visits, and to illustrate differences between those who returned and those who did not. Overall, there is a tendency for greater percentage returns with each successive visit. At each wave, those who were quit at a given wave were more likely to return for a subsequent visit, with the percentage quit increasing at each visit, whereas the percentage using the QuitCoach to make a new attempt decreased. Relapsers, in particular, were less likely to return. While the percentage quit increased (at least up to visit 5), most of the quitting took place between the first and second wave. By wave 4, those who returned were marginally less likely to be quit at the next wave, largely due to an increase in new attempts. The percentage using to make a subsequent attempt (more than a week post-relapse) increased over time (4.8% at visit 2 to 8.9% at visit 5), while the percentage using it to recover from a relapse (within a week) declined from 14.2% at visit 2 to 4.3% at visit 5. Among smokers at wave 2, those who had relapsed within the last week were less likely to return for a third visit than those who had not made any quit attempt (26.4% vs 38.8%, χ^2^_1_= 21.13, *P* < .001).

By wave 5, over half of active users had successfully quit for more than a month, and less than 1% had failed to make any quit attempt.

### QuitCoach Use for Recovery From Relapse

Recent relapse reported on one visit was associated with reduced subsequent use. The 11.4% of smokers coming to the site initially who had relapsed from a previous quit attempt within a week of their initial visit were less likely than other users to return for subsequent visits (22.9% cf 27.2%). Of those who did return (n = 620), 52.1% were quit at visit 2 and another 28.7% had tried again but relapsed. Overall, 38.9% of this group who made a second visit returned for a third, with those now quit at visit 2 more likely to do so (46.7% vs 27.0% for double relapsers and 35.3% for those who did not try to quit again between visits). Of those smoking at visit 2 who returned (n = 90), 41.1% were now quit and 34.4% had tried (again) and failed. Of those quit at visit 2 (n = 151), 84.8% were still quit at visit 3. A similar pattern was found for the 14.2% of the sample (34.1% of smokers at time 2) who were recent relapsers at their second visit. Those in this group were less likely to return for a third visit (23.3% cf 32.5%), and of those who did return (n = 214), 41.6% were quit at visit 3 and another 41.1% had tried again but relapsed.

**Table 3:** Cessation activity between visits

Status at visit		First to second			Second to third			Third to fourth			Fourth to fifth		
		Status at V1		Status at V2	Status at V2		Status at V3	Status at V3		Status at V4	Status at V4		Status at V5
N		6461		6461	2714		2714	1062		1062	583		583
% of previous wave				27.0			42.0			39.1			54.9
% of total sample				27.0			11.5			4.5			2.5
Average interval (days)			19.3			22.8			28.6			36.2	
**To prevent relapse**													
	Quit since last visit	N/A		53.4	64.2		11.8	13.5		5.6	6.2		3.9
	Quit before last visit												
	< 1 month	4.8^a^		3.5	5.0		52.1	57.8		38.7	44.6		27.6
	≧ 1 month	1.3^a^		2.0	2.1		13.1	12.0		38.3	37.2		54.4
Total Quit		6.1		58.9	71.3		77.0	83.3		82.6	88.0		85.9
**To recover from relapse**													
	Relapse < 1 week ago	9.5		14.2	7.9		9.3	5.7		6.2	5.3		4.3
**To make a quit attempt**													
	Relapse ≧ 1 week ago	15.9		4.8	2.9		4.3	2.8		5.6	3.1		6.3
	Failed quit before last visit	N/A		N/A	N/A		2.4	1.7		3.2	1.4		2.6
	New quit attempt	15.9		4.8	2.9		6.7	4.5		9.0	4.5		8.9
	No previous quit attempts	68.4		22.0	17.8		7.0	6.5		2.4	2.2		0.9
Total use to make a new attempt on that wave		84.3		26.8	20.7		13.7	11.0		11.2	6.7		9.8

^a^First visit refers to time quit, as there is no previous visit.

## Discussion

The QuitCoach appears to attract a diverse range of smokers. There is a predominance of females, and less surprisingly, a relatively young age profile. Men and older smokers are also less likely to use other forms of behavioral cessation assistance such as Quitlines [20,21]. Balmford et al [18] show that in relation to the general population of Australian smokers, QuitCoach users are also slightly more addicted and more likely to have made a recent (failed) quit attempt.

Most QuitCoach users start using the program as smokers; that is, they mainly come to the site to help them quit. If they persist with the program (and only a minority do), they predominantly do so to help themselves stay quit. This also includes higher rates of return use among those few who started using when quit. There are also small but important minorities who use the site to initiate a subsequent quit attempt, both immediately after a failed initial attempt and after some delay (use beyond the prompting period), and some who use it to recover from a relapse. The QuitCoach is designed to support all three of these types of use.

Users are much more likely to continue using to stay quit than recover from relapses or initiate new attempts. Similarly, Wang and Etter [22], in a real-world evaluation of an online, tailored advice program with email prompts, found users in the action stage to be most likely to return. This probably reflects the reality that most smokers give up for a while after setbacks in a quit attempt, waiting for some time before they are prepared to try again. That there is any repeated use by those struggling to overcome obstacles is particularly gratifying, assuming the use is helpful.

The analyses reported here all assume that the information users provide is accurate. Most of the responses are consistent (some of this may be due to logic checks effectively forcing consistency); however, there are a small percentage of apparently inconsistent responses. For example, among users who were smoking at baseline but quit at visit 2, a number reported being quit for longer than their inter-visit interval. We suspect that there is some use of the site for “what if” purposes, exploring what advice would be provided if the questions had been answered differently. In this case, what they might expect if quit for longer. Because the advice provides normative information as to what is typically experienced at various points in the quitting process, it would be potentially useful to find out what to expect in the future. Otherwise the patterns of responses are consistent, insofar as we have analyzed them, suggesting most users report their current situation and respond consistently.

We expected repeat usage of the program to be greater among those with greatest need; however, the evidence for this was mixed. The lowest rate of return was in the lowest dependence group, and very rapid return was more frequent for those most dependent, suggesting that a proportion are using in ways consistent with probable need. However, those with a longer previous quit attempt were more likely to return, as were those using pharmacotherapy and those using other professional help. This suggests that some of the repeated use is from those who are help seekers by nature, not necessarily those who might need it most. Women and older smokers were more likely to return, as has been found for a similar online program [22].

This study cannot be used to assess the effectiveness of the program. However, it has been shown to be effective when delivered in a different manner [14]. That said, it is apparent that a minority of users have developed an ongoing relationship with the site, consistent with them at least perceiving considerable benefit. Moreover, those continuing to use once quit achieved high rates of abstinence, at least over the period they continued to visit.

Rates of initial use by those already quit were low. The proportion of first-time users who were already quit was considerably lower than has been found for the Victorian Quitline, a telephone-based support service targeted to the same population of smokers provided by Quit Victoria [18,23]. Both the finding that initial use is almost exclusively by smokers, and that those first using when quit were more likely to continue using, suggests that most users coming to the site believe that quit smoking advice is primarily something that is useful before quitting. Without the experience of getting advice and seeing that it can apply to the post-quitting period, they may not spontaneously see the need for it or fail to understand the capacity of an online expert system to tailor information to those already quit. That those returning for all subsequent visits were more likely to be quit at previous visits is consistent with this explanation. Finding effective ways to encourage recent quitters who are experiencing difficulties to seek help is a priority. There is a need to better inform smokers of the capacity of online programs such as this to deliver interactive tailored advice and that tailored advice can be generated regularly throughout the quitting process to facilitate both a quit attempt and staying quit.

Only 27% of registered users (31% more recently) returned for a second assessment, despite prompting to return by up to two reminder emails each. Failure to return to an Internet-based smoking cessation program is common. Wang and Etter [22], for example, reported that only 20% of users of the Swiss Stop-Tabac program returned for a second visit when prompted by email. Moreover, Saul et al [24] achieved only a 39.4% online follow-up rate when users of a Web-based cessation intervention were actively followed up 6 months after initial use. The QuitCoach did not actively follow up with users, nor explicitly request that they return. Rather, the emails simply suggested to users that it would be a good time for them to return for a re-assessment, as things may have changed and the advice they received last time may no longer be relevant. That most returns to the site occurred soon after receipt of a reminder email suggests that they were having some effect.

There are several possible reasons why most users failed to return. Some may have lost interest in quitting altogether; others may have believed that they were doing so well that they didn’t need any more help; and still others may not have progressed and saw no need as they might have expected the advice they received would be largely identical to what they had previously received. We may have failed to remind others because of a changed email address (or because an incorrect address was deliberately provided due to privacy concerns) [25], because our reminder was blocked by a spam filter, or because the email was perceived to be spam [26].

The program also may not have been effective in communicating the value of returning. Findings from a series of user-based site evaluations we conducted in 2006 suggest that many users simply did not understand why they should return. Participants in the evaluation consistently read their advice with great interest and commented favorably on it; yet some expressed surprise when asked whether they would return. As they had already received useful quitting advice, it was not apparent to them why they would need to return for more. We have taken steps to redress this by providing better information on what the site offers and how to use it, including a greater emphasis on its potential value post-quitting. Moreover, the program needs to do more to build the kind of relationship with a smoker that will foster ongoing interaction, in part by signaling the value of this relationship. We are exploring the use of SMS messaging (or other mobile phone-delivered media such as MMS) to provide timely prompts and reminders to help smokers manage and remain engaged with their quit attempt over time and to return to the site at strategically important points. Messages delivered in this medium are being designed to provide brief snippets of information and advice tailored to the user’s perspective on change and to other potentially important predictor variables that are measured during the QuitCoach assessment. SMS messaging has been shown in one trial to be an effective way to deliver smoking cessation support [27], although that program was not designed to be integrated with a program with the capacity for more detailed advice provision.

The other challenge is to maintain longer-term interest in quitting among those who fail. It is gratifying that some, albeit a small percentage, seem to return spontaneously when engaged in a subsequent quit attempt. This demonstrates that these smokers at least saw value in the advice and were prepared to use the program again. As smoking cessation often takes several attempts, programs such as this that one can return to are likely to be of benefit to some.

Personalized, tailored cessation advice programs like the QuitCoach have the potential to reach many smokers very economically. The QuitCoach has been used by many, but rarely to the extent thought optimal. More research is needed both on the marginal additional benefits of additional assessment/feedback cycles for those who currently choose not to use them, and on ways of optimizing use.

## Abbreviations

HSI-AV: heaviness of smoking index—alternate version
IQR: inter-quartile range
MMS: multimedia messaging service
NRT: nicotine replacement therapy
RCT: randomized controlled trial
SMS: short message service
